# Supplementary material for: Transcriptome profiling of histone writers/erasers enzymes across spermatogenesis, mature sperm and pre-cleavage embryo: Implications in paternal epigenome transitions and inheritance mechanisms
Source: Front Cell Dev Biol. 2023 Jan 27;11:1086573. doi: 10.3389/fcell.2023.1086573 (PMC9911891; doi:10.3389/fcell.2023.1086573)
Supplement: Supplementary file 1 [file DataSheet1.PDF]

Supplemental information

Dataset validation

To validate the GSE dataset, we evaluated consistent markers expression across cell types (Fig. S1). SGund showed peak expression of stem cell markers *Thy1*, *Nanos2*, *Nanos3*, *Gfra1*, *Zbtb16* and *Uchl1* and SGdiff the committed spermatogonia marker *Kit*. As it is established, the transition from SGund to SGdiff coincides with the gain of the c-kit receptor in the adult testis. Kit continues to be expressed until meiosis and plays essential roles in the survival of SGdiff. The PreL stage showed shared expression with SGdiff gene *Kit* and LZ stage genes *Dazl* and *Top2a*. LZ stage showed differentiation markers *Stra8* and *Sycp1/2/3*, whereas PD stage showed meiosis markers *Spo11*, *Piwil* and *Pttg1*. The PD stage shared gene expression with RStid of *Ddx4*, *Dkkl1*, *Insl6*, *Acrbp* and *Acr*, and RStid stage also showed peak expression of classical spermiogenesis markers *Tnp1/2* and *Prm1/2*, and *Izumo1*, *Spag6*, *Acrv1* and *Pgk2*. To check the germ cells purity, we analyzed marker genes of other cell populations in the testis including Leydig (*Cyp11a1*, *Cyp17a1*, *Hsd3b1* and *Star*), Sertoli (*Sox9*, *Drd4* and *Rhox8*), endothelial (*Vwf* and *Tie1*), myoid (*Acta2* and *Myh11*) and machophage (*Adgre1*) cells, which were detected in very low levels (supplemental Table 1).

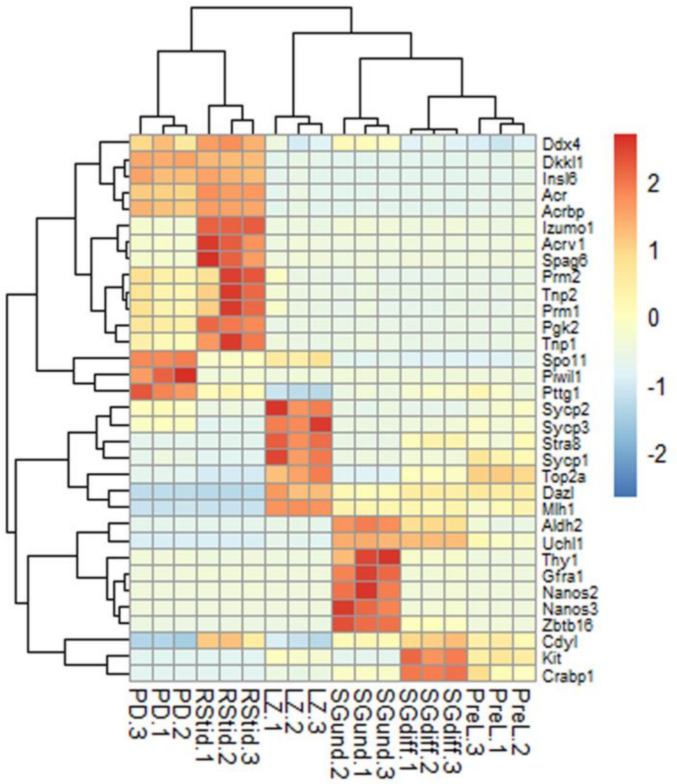

Figure S1: Unsupervised hierarchical clustering analysis of male germ cells transcriptome. Heatmap showing z-score values of developmental markers. SGund: spermatogonia Kit-, SGdiff:

spermatogonia Kit+, PreL: pre-leptotene spermatocytes, LZ: leptotene/zygotene spermatocytes, PD: pachytene/diplotene spermatocytes, RStid: round spermatid.

Downregulated enzymes across spermatogenesis

We analyzed downregulated mRNAs of epigenetic enzymes across male germ cells differentiation, performing differential gene expression (DEG) analysis using the DESeq2 algorithm, and contrasts on consecutive stages of cell development: PreL vs SGdiff, LZ vs PreL, PD vs LZ and RStid vs PD ($padj<0.05$, $log2FC<-0.5$, see Fig. S2).

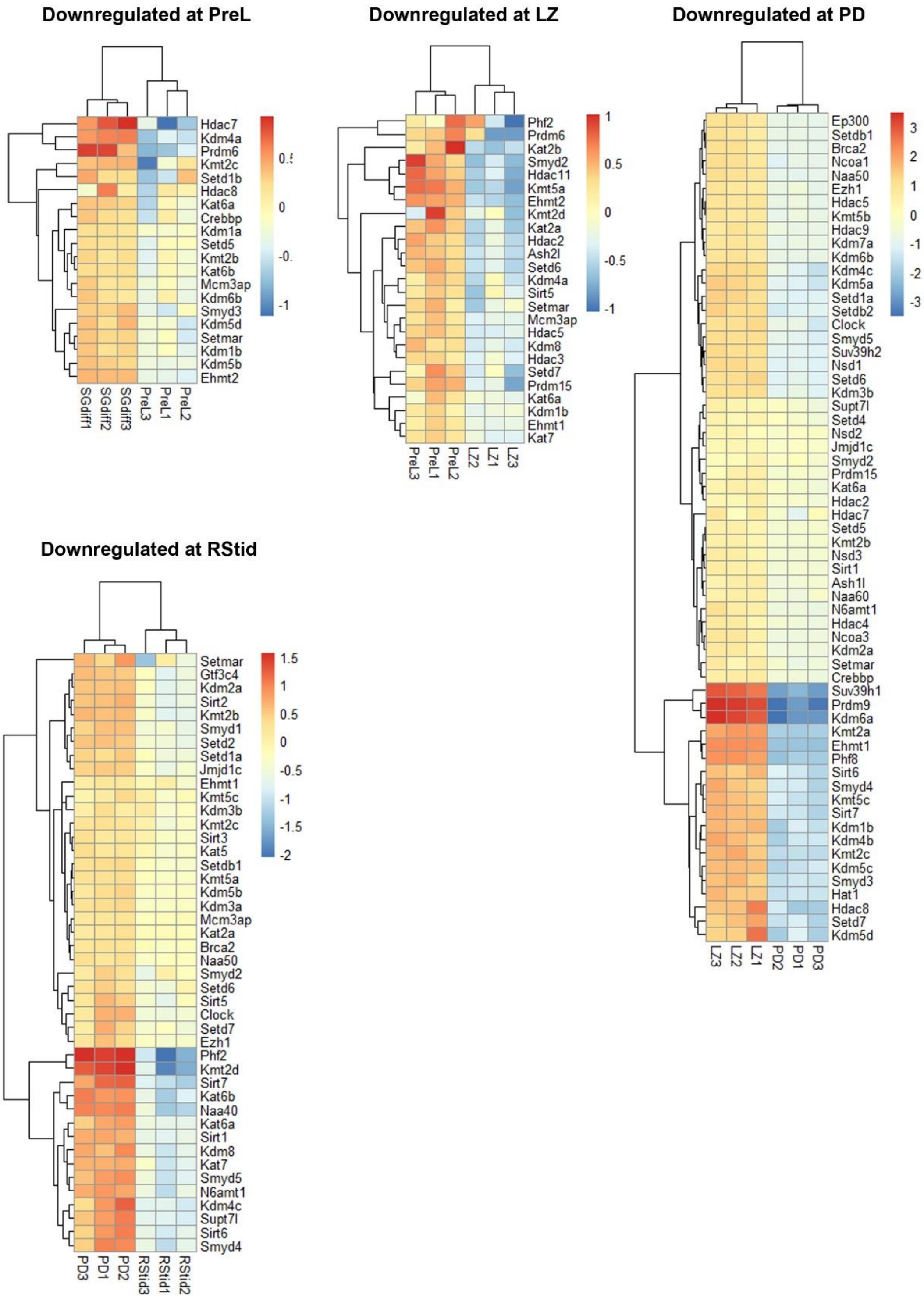

Figure S2: Heatmaps showing z-score values for each germ cell stage. Differential gene expression analysis was conducted with DESeq2 with LRT test and contrasts on consecutive populations across differentiation (PreL vs SGdiff, LZ vs PreL, PD vs LZ and RStid vs PD). Downregulated genes were selected with padj<0.05 and log2FC<-0.5.

Data cross validation

To perform data cross validation, we compared the expression profile of the GSE162740 (Mayorek et al., 2022) with two more datasets:

- ✓ GSE112393 (Green CD, Ma Q, Manske GL, et al. A Comprehensive Roadmap of Murine Spermatogenesis Defined by Single-Cell RNA-Seq. Dev Cell. 2018, 46(5):651-667.e10. doi:10.1016/j.devcel.2018.07.025): this dataset contains single cell RNAseq data, that characterized 12 germ cell clusters in the adult mouse testis: spermatogonia (SG: GC1), preleptotene (Prelep: GC2, GC3), spermatocytes (SCyte: GC4, GC5, GC6, GC7, GC8), spermatids (STid: GC9, GC10, GC11) and elongated spermatids (eSTid: GC12). We used the mean log2+1 expression.

- ✓ GSE49622 (Hammoud SS, Low DH, Yi C, Carrell DT, Guccione E, Cairns BR. Chromatin and transcription transitions of mammalian adult germline stem cells and spermatogenesis. Cell Stem Cell. 2014;15(2):239-253. doi:10.1016/j.stem.2014.04.006): this datasets contains RNAseq data from spermatogonia positive for the undifferentiated state marker Thy1 (SGThy1, N=1) and for the committed marker Kit (SGKit, N=1), paquitene/diplotene spermatocytes (PD, N=5) and spermatids (Stid, N=5). We mapped the FASTQ files with the same workflow used for the Mayorek dataset, and calculated the RPKMs.

For each dataset we extracted the enzymes expression and performed hierarchical clustering (pheatmap package), defining clusters with strong population tendencies.

As it is shown in Figure S3, column clustering of the Green dataset shows that the spermatogonia (SG) forms a separate population, followed by preleptone cells (PreL), and then 4 clusters composed of early (SCytes1) and late (SCyte2) spermatocytes, the round spermatids (STid) and the elogated spermatids (eSTid). Gene expression grouped in 9 clusters, with different peak expression across cell populations.

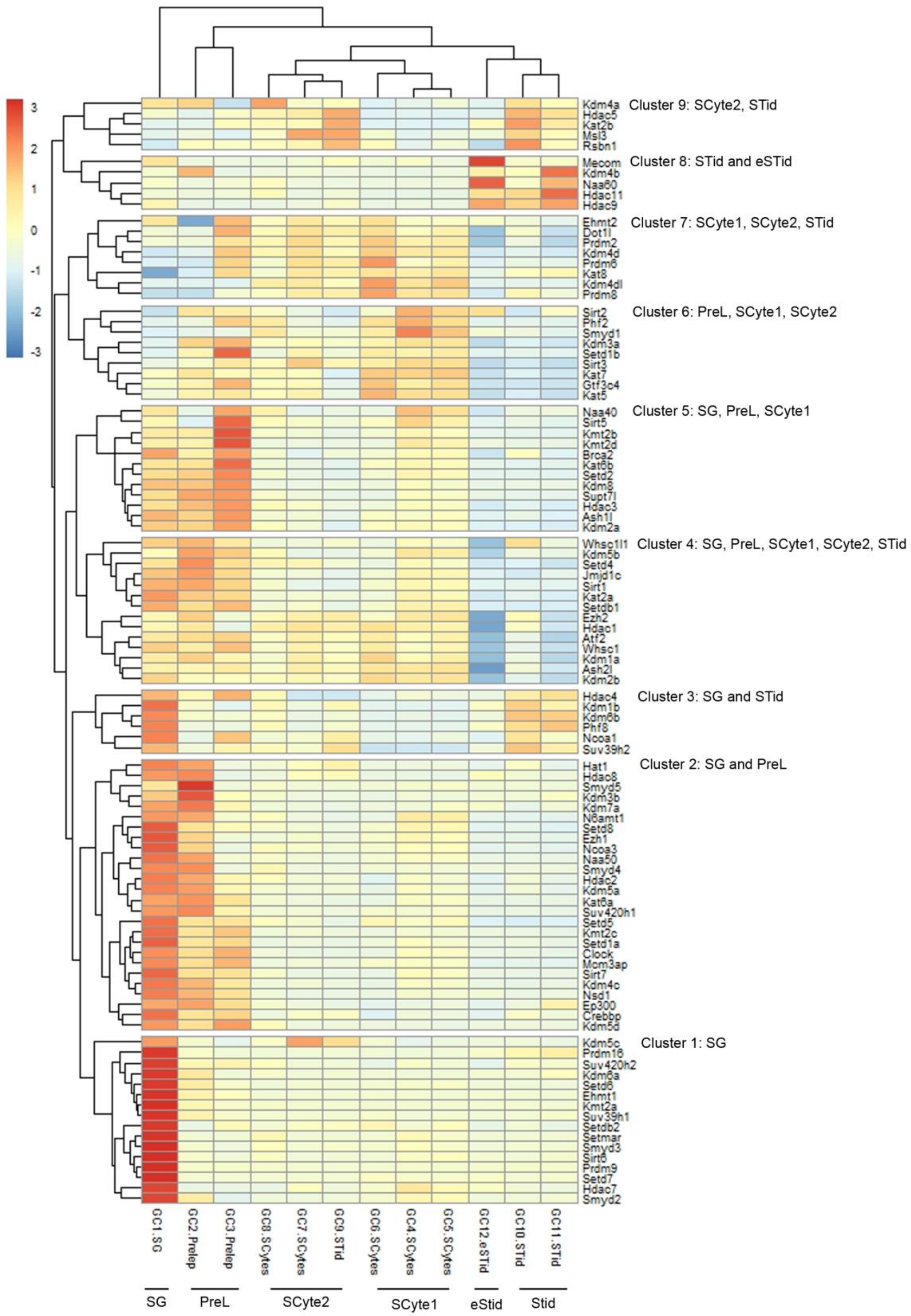

Figure S3: Hierarchical clustering of z-scores of mean expression from Green et al., 2018 single cell RNAseq dataset.

As it is shown in Figure S4, column clustering of the Hammoud dataset shows 3 main clusters composed of spermatogonia (SG), paquitene/diplotene spermatocytes (PD) and spermatids (STid). Although there seems to be differences within the SG population, as some genes seem higher in SGThy or SGKit, we cannot confidently assign expression to one or the other because of lack of replicas, hence we considered both SG samples as replicas (which is also supported by the column clustering). Gene expression grouped in 7 clusters.

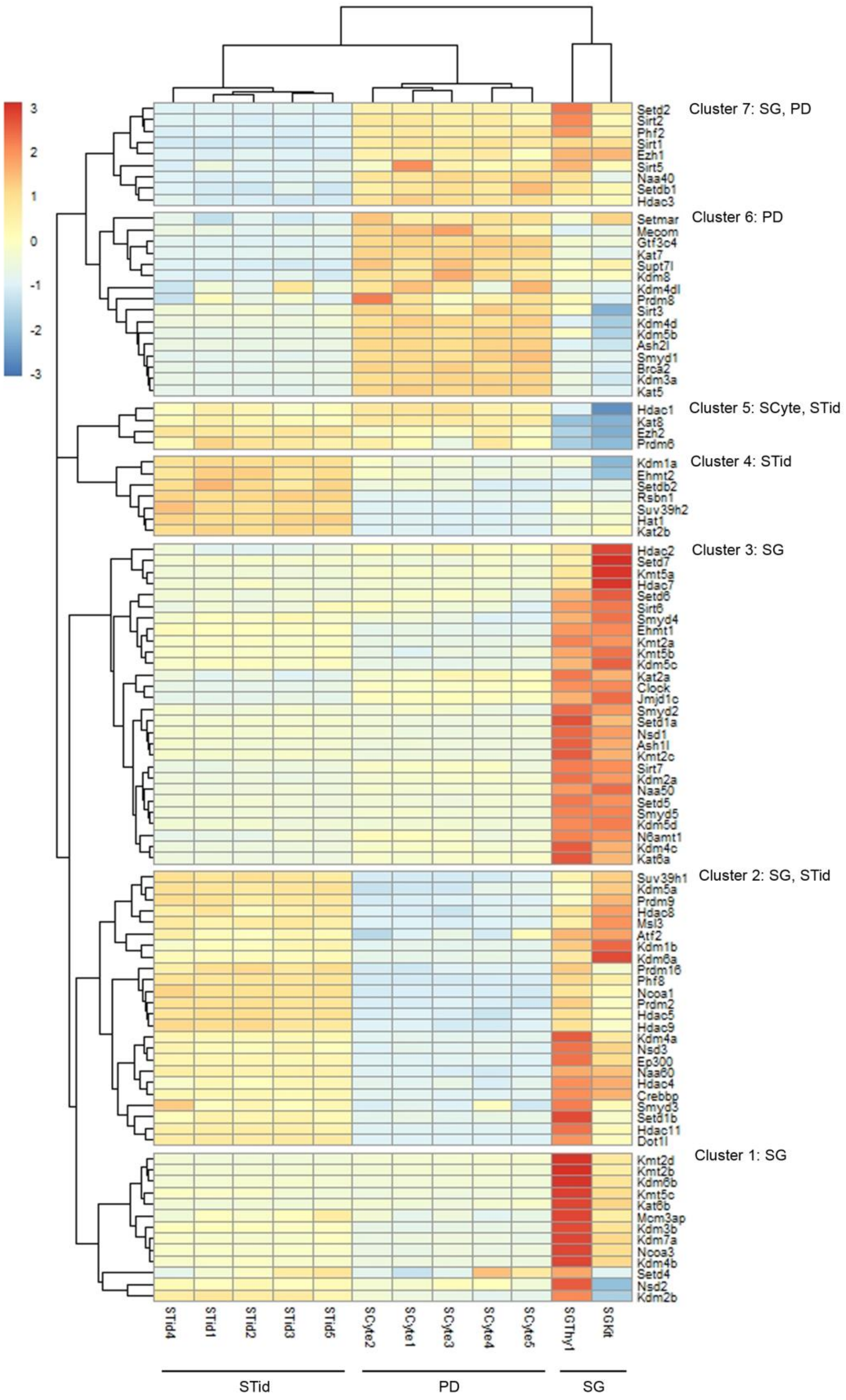

Figure S4: Hierarchical clustering of z-scores of RPKMs from Hammoud et al. 2014 RNAseq dataset.

Finally, we constructed a correspondence table between the three datasets (Table S5), following this criteria:

- SGund and SGdiff was validated when expression was also positive in SG from Green and/or Hammoud
- PreL was validated when expression was also positive in PreL from Green
- LZ was validated when expression was also positive in SCyte1from Green
- PD was validated when expression was also positive in SCyte2 from Green and/or PD from Hammoud
- RStid was validated when expression was also positive in Stid/eStid from Green and/or STid from Hammoud

Following this approach we labeled each enzyme upregulated at each developmental stage in the Mayorek dataset as follows:

(green)	match developmental stage with Green and Hammoud
(orange)	match developmental stage with Green
(blue)	match developmental stage with Hammoud
(yellow)	match with a close developmental stage with Green and/or Hammoud

Table S5: correspondence table of Mayorek dataset with Green single cell RNAseq and Hammoud RNAseq datasets. * increased expression from previos stage padj < 0.05 and log2FC > 0.5. X: expression assigned from clustering of z-score.

	Mayorek RNAseq						Green single cell RNAseq							Hammoud RNAseq			
Enzyme	SGund	SGdiff	Prel	LZ	PD	RStid	cluster	SG	Prel	SCyte1	SCyte2	STid	eStid	cluster	SG	PD	STid
Hdac4	*			*			3	x				x		2	x		x
Kdm5b	*				*		4	x	x	x	x	x		6		x	
Smyd3	*			*			1	x						2	x		x
Setd7	*		*				1	x						3	x		
Kdm4a	*				*	*	9				x	x		2	x		x
Kmt2c	*			*			2	x	x					3	x		
Supt7l	*			*			5	x	x	x				6	x	x	
Kdm2b	*			*			4	x	x	x	x	x		1	x		
Setd1b	*				*		6		x	x	x			2	x		x
Smyd1	*				*		6		x	x	x			6		x	
Kdm3a	*		*	*	*		6		x	x	x			6		x	
Smyd5	*						2	x	x					2	x		
Hdac11	*					*	8					x	x	2	x		x
Setmar	*						1	x						6	x	x	
Setd5	*			*			2	x	x					3	x		
Kat6a	*						2	x	x					3	x		
Jmjd1c	*						4	x	x	x	x	x		3	x		
Kat2a	*				*		4	x	x	x	x	x		3	x		
Ezh1	*			*			2	x	x					7	x	x	
Hdac9	*		*			*	8					x	x	2	x		x
Kdm1b	*					*	3	x				x		2	x		x
Phf2	*		*		*		6		x	x	x			7	x	x	
Ep300	*			*			2	x	x					2	x		x
Hdac7	*						1	x						3	x		
Kmt2d	*				*		5	x	x	x				1	x		
Crebbp	*					*	2	x	x					2	x		x
Setd4	*					*	4	x	x	x	x	x		1	x		
Kat2b	*				*	*	9				x	x		4			x
Kdm4b	*			*		*	8					x	x	1	x		
Kdm3b	*						2	x	x					1	x		
Suv39h1	*		*	*		*	1	x						2	x		x
Hdac8	*			*		*	2	x	x					2	x		x
Kdm5c	*		*			*	1	x						2	x		
Msl3	*		*		*	*	9				x	x		2	x		x
Kdm5d	*					*	2	x	x					3	x		
Suv39h2		*				*	3	x				x		4			x
Ehmt1		*					1	x						3	x		
Kdm4c		*	*				2	x	x					3	x		
Hdac1		*			*		3	x	x	x	x	x		5		x	x
Kmt5a		*	*				2	x	x					3	x		
Kdm4d		*	*	*	*		7		x	x	x	x		6		x	
Sirt1		*					4	x	x	x	x	x		7	x	x	
Kdm6b		*					3	x				x		1	x		
Ncoa1		*	*			*	3	x				x		2	x		x
Setdb2		*	*	*		*	1	x						4			x
Prdm9		*	*	*		*	1	x						2	x		x
Hdac3		*					5	x	x	x				7	x	x	
Kmt5c			*	*			1	x						3	x		
Sirt7			*	*			2	x	x					3	x		
Sirt2			*	*	*		6		x	x	x			7	x	x	
Sirt6			*				1	x						3	x		
Clock			*				2	x	x					3	x		
Kdm7a			*				2	x	x					1	x		
Phf8			*	*		*	3	x				x		2	x		x
Nsd3			*	*		*	4	x	x	x	x	x		2	x		x
Atf2			*			*	4	x	x	x	x	x		2	x		x
Hdac5			*			*	9				x	x		2	x		x
Sirt5			*		*		5	x	x	x				7	x	x	
Ezh2			*			*	4	x	x	x	x	x		5		x	x
Setd6			*				1	x						3	x		
Kdm6a			*	*		*	1	x						2	x		x
Prdm8			*	*	*		7		x	x	x	x		6		x	
Kdm4dl			*	*	*		7		x	x	x	x		6		x	
Gtf3c4				*	*		6		x	x	x			6		x	
Nsd1				*			2	x	x					3	x		
Naa50				*			2	x	x					3	x		
Ncoa3				*			2	x	x					1	x		
Setd1a				*			2	x	x					3	x		
Kdm2a				*			5	x	x	x				3	x		
Kdm5a				*			2	x	x					2	x		x
Hat1				*		*	2	x	x					4			x
Smyd4				*			2	x	x					3	x		
N6amt1				*			2	x	x					3	x		
Brca2				*			5	x	x	x				6		x	
Setdb1				*			4	x	x	x	x	x		7	x	x	
Hdac1					*		4	x	x	x	x	x		7		x	x
Dot1l					*	*	7		x	x	x	x		2	x		x
Kdm1a					*		4	x	x	x	x	x		4			x
Naa40					*		5	x	x	x				7	x	x	
Mecom					*		8					x	x	6		x	
Kat7					*		6		x	x	x			6		x	
Sirt3					*		6		x	x	x			6		x	
Kat8					*		7		x	x	x	x		5		x	x
Ehmt2					*	*	7		x	x	x	x		4			x
Rsbn1					*	*	9				x	x		4			x
Ash2l					*		4	x	x	x	x	x		6		x	
Prdm2					*		7		x	x	x	x		2	x		x
Kat5					*		6		x	x	x			6		x	
Prdm6					*	*	7		x	x	x	x		5		x	x
Nsd2						*	4	x	x	x	x	x		1	x		
Prdm16						*	1	x						2	x		x
Smyd2	*	*	*				1	x						3	x		
Kdm8	*	*	*				5	x	x	x				6	x	x	
Hdac2	*	*	*				2	x	x					3	x		
Mcm3ap	*	*	*				2	x	x					1	x		
Setd2	*	*	*	*	*		5	x	x	x				7	x	x	
Kat6b	*	*	*	*	*		5	x	x	x				1	x		
Kmt2b	*	*	*	*			5	x	x	x				1	x		
Naa60	*	*	*	*			8					x	x	2	x		x
Kmt5b	*	*	*	*			2	x	x					3	x		
Ash1l	*	*	*	*			5	x	x	x				3	x		
Kmt2a	*	*	*	*			1	x						3	x		

As it is shown in Table S6, using this approach we were able to validate 77.14 % of our data with high match between the two datasets in the shared populations. Also, 8.1% of the data was observed in a consecutive developmental stage, and 14.8% were exclusively detected in the Mayorek dataset.

Table S6: sum and percentage obtained for match with Green and Hammoud (green), match with Green (orange), match with hammoud (blue), match with a close developmental stage (yellow) and no match (white).

**sum**	86	55	21	17	31	**210**
**percent**	41.0	26.2	10.0	8.1	14.8	**100**
	**77.14**					
